# Impact of schizophrenia relapse definition on the comparative effectiveness of oral versus injectable antipsychotics: A systematic review and meta‐analysis of observational studies

**DOI:** 10.1002/prp2.915

**Published:** 2022-01-28

**Authors:** Tiffany Cristarella, Genaro Castillon, Jean‐François Nepveu, Yola Moride

**Affiliations:** ^1^ Faculty of Pharmacy Université de Montréal Montreal Quebec Canada; ^2^ YolaRX Consultants Montreal Quebec Canada; ^3^ Department of Epidemiology, Biostatistics and Occupational Health McGill University Montreal Quebec Canada; ^4^ Rutgers The State University of New Jersey New Brunswick New Jersey USA; ^5^ Present address: Institute for Health Health Care Policy and Aging Research Rutgers The State University of New Jersey 112 Paterson St New Brunswick New Jersey 08901 USA

**Keywords:** comparative effectiveness, long‐acting injectable antipsychotics, meta‐analysis, oral antipsychotics, schizophrenia relapse, systematic review

## Abstract

Although relapse is an important outcome to measure the effectiveness of schizophrenia treatment, no standard definition exists. This review aimed at identifying definitions and measurements of schizophrenia relapse in observational studies of long‐acting injectables (LAIs) versus oral antipsychotics (OAPs) and at determining their impact on heterogeneity of comparative effectiveness estimates. A systematic review was conducted using MEDLINE and Embase (01 January 2010–11 November 2019 [date last searched]). Pragmatic searches of gray literature and snowballing were also conducted. Search outputs were screened independently by two assessors at first stage, and full‐text of potentially eligible sources at second stage. For each retained source, definition and measurement of relapse, study methods, and comparative effectiveness estimates were extracted. Heterogeneity of estimates was assessed using *I* ^2^ statistic with a threshold of 50% for substantial heterogeneity. Literature search yielded 543 sources and pragmatic searches, 21, of which 35 were eligible. Twelve definitions of relapse were found based on hospitalization/emergency department (ED) data (28 studies) or clinical assessment (5 studies). No definition was provided in five studies. According to quantitative analyses, in studies defining relapse as schizophrenia‐related hospitalization and/or ED visits over 1‐year follow‐up, LAIs were significantly more effective than OAPs. For studies measuring relapse based on all‐cause hospitalization, heterogeneity was too high for pooling; yet this definition is the most frequently found in pooled estimates published in the literature. Schizophrenia relapse definitions led to substantial heterogeneity of comparative effectiveness estimates of LAIs versus OAPs. Creating study subgroups based on relapse definition effectively reduces statistical heterogeneity.

## Graphical abstract

Definition of schizophrenia relapse is a major source of heterogeneity in observational studies comparing long‐acting injectables with oral antipsychotic drugs. Hence, standardization of criteria to ascertain relapse as an outcome would be needed to allow synthesis of available findings.

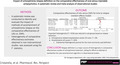

**Abbreviations**
CGI‐S: Clinical Global Impression Scale
CI: confidence interval
ED: emergency department
EMR: electronic medical record
HR: hazard ratio
ICD: International Classification of Disease
IOM: Institute of Medicine
LAI: long‐acting injectable
NAM: National Academy of Medicine
OAP: oral antipsychotic
OR: odds ratio
PICOTS: population, intervention, comparator, outcomes, timing, setting
PRISMA: preferred reporting items for systematic review and meta‐analysis statement
RR: relative risk
US: United States
VA: Veterans Affairs

## 1 INTRODUCTION

Schizophrenia is a chronic disabling disorder that affects 1% of the world population.^1^ Most patients with schizophrenia experience multiple relapses, characterized by worsening of psychotic symptoms leading to progressive cognitive deterioration, impaired functioning, hospitalizations, greater risk of suicide, and reduced quality of life.^1^, ^2^, ^3^, ^4^ Treatment nonadherence to oral antipsychotics (OAPs), ranging from 40% to 50% and even as high as 89%,^5^, ^6^ is a known barrier to treatment success in this population^7^ and a major contributor to relapse.^8^ For patients who are not adherent to OAP treatment, clinical guidelines recommend long‐acting injectables (LAIs).^5^ Although it is recognized that LAIs are associated with more favorable treatment adherence than OAPs,^7^, ^8^ observed benefits on clinical outcomes, such as relapse, are conflicting.^9^ According to a systematic review of 87 observational studies,^1^ schizophrenia relapse is a complex condition for which a patient may relapse without the need for hospitalization such as experiencing a moderate symptom exacerbation; therefore, there are currently no established criteria used to define schizophrenia relapse.

It is hypothesized that the definition of relapse is a source of heterogeneity across comparative effectiveness studies of LAIs versus OAPs, thereby hampering the pooling of estimates through meta‐analysis. Furthermore, the handling of death in studies (i.e., exclusion criterion, censoring criterion, or not considered) may further contribute to the heterogeneity as relapse may be associated with suicide‐related mortality in this population.^10^

This study aimed at identifying the definitions and measurements of schizophrenia relapse used in observational studies of LAIs versus OAPs, and at determining their impact on the heterogeneity of comparative effectiveness estimates.

## 2 MATERIALS AND METHODS

### 2.1 Study design

A systematic review was conducted and reported according to the preferred reporting items for systematic review and meta‐analysis (PRISMA) statement.^11^ Protocol was registered in PROSPERO under CRD42020162054.

### 2.2 Information sources

The literature search was conducted in Ovid MEDLINE and Embase using free‐text keywords and thesaurus terms (i.e., MeSH and Emtree terms, respectively for MEDLINE and Embase). Search period covered 01 January 2020 until 11 November 2019, date last searched. Search of the databases was in English, but outputs published in English, French, or Spanish were considered.

Pragmatic searches involved web searches using Google (www.google.com/) and Google Scholar (www.google.com/scholar/) search engines as well as the OpenGrey database. In addition, websites of relevant learned societies were searched in December 2019 using the keywords “schizophrenia relapse.” The lists of references of retained sources and reviews were also hand‐searched for additional sources (snowballing).

### 2.3 Study selection

**FIGURE 1:**
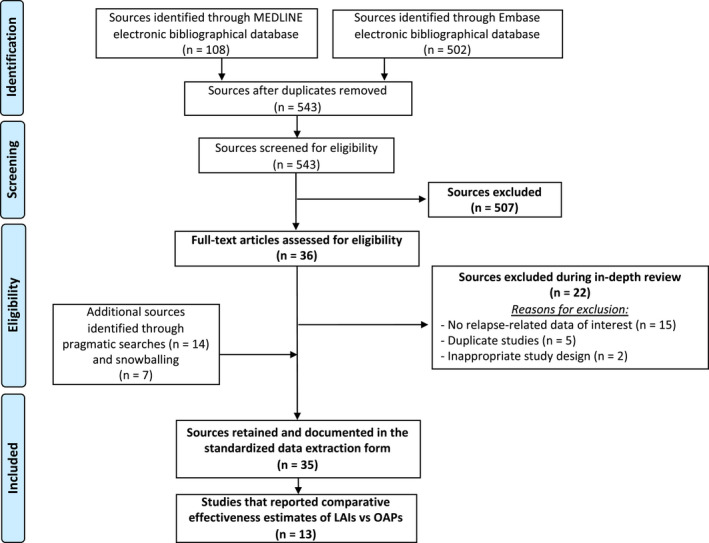
PRISMA flowchart of searches on the comparative effectiveness of LAIs versus OAPs in preventing relapse in schizophrenia

Search outputs were uploaded into EndNote X9 and duplicates were deleted. In phase 1, sources were screened independently by two reviewers (with conflicts resolved by a third), followed by an in‐depth review of retained sources to confirm eligibility (phase 2). Study selection is presented in a PRISMA flowchart (Figure 1).^11^

### 2.4 Eligibility criteria

The PICOTS criteria are as follows: *Population*: Patients diagnosed with schizophrenia; *Intervention*: LAIs; *Comparator*: OAPs; *Outcomes*: Relapse definition and measurement, death handling (i.e., censoring criterion, exclusion criterion, study outcome [all cause or psychiatric‐related]), handling of loss to follow‐up (i.e., censoring criterion, exclusion criterion, unspecified), and comparative effectiveness estimates of LAIs versus OAPs on relapse (relative risk [RR], odds ratio [OR], hazard ratio [HR] and 95% confidence interval [CI]); *Time period*: 01 January 2010–11 November 2019 (date last searched); *Setting*/*Study types*: Inpatient or outpatient/Observational (noninterventional) studies.

Case reports, case series, expert opinions, editorials, Phase I–III clinical trials, and nonclinical studies were excluded.

### 2.5 Extracted data

Data extraction was conducted by a single reviewer, and the data extraction form was first piloted by two independent assessors (with conflicts resolved by a third). In addition to the outcomes of interest, data extracted included study design, data source, sample size, follow‐up duration, patient characteristics (age and sex distribution, ethnicity, comorbidities), number of patients treated with either OAPs or LAIs, and treatment names. No attempt was made to contact the study authors for additional data. The review was conducted according to methods proposed by the Cochrane group^12^ and the Institute of Medicine (IOM) of the National Academy of Medicine (NAM).^13^

### 2.6 Synthesis of evidence (including meta‐analysis)

The subset of studies that reported comparative effectiveness estimates of LAIs versus OAPs were retained for the assessment of heterogeneity. First, a qualitative assessment of methodological and clinical heterogeneity was undertaken based on study design (risk estimate and follow‐up duration) and study populations (age and sex distributions). If two or more studies were clinically and methodologically homogeneous, statistical heterogeneity of estimates was quantified using *I* ^2^ statistic, which is the percentage of variation across studies due to heterogeneity rather than chance. When overall heterogeneity exceeded 50%,^14^ studies were stratified by relapse definition. An *I* ^2^ was calculated for each subgroup, and if within the acceptable range, comparative effectiveness estimates were pooled using the inverse variance method with a random effects model.^15^ Pooled estimates and corresponding 95% CIs were obtained using the Review Manager Software (RevMan Version 5.3. Copenhagen: The Nordic Cochrane Centre, The Cochrane Collaboration, 2014). The assessment of publication bias through a funnel plot asymmetry test (Egger's test) was planned. However, this was deemed unfeasible as the number of studies retained in each subgroup was less than 10, which is the minimum required.^16^ A methodological quality assessment of individual studies was not undertaken.

## 3 RESULTS

### 3.1 Search results

The literature search yielded 543 sources, of which 36 were retained for in‐depth review. There were 22 sources that were further excluded at stage 2 for reasons shown in Figure 1. Pragmatic searches and snowballing yielded 21 additional sources. Data were thus abstracted from 35 eligible sources (31 full‐text publications and 4 abstracts). Of these, 13 reported comparative effectiveness estimates of LAIs versus OAPs; the others described the population with relapse without studying treatment effects.

### 3.2 Definitions and measurements of schizophrenia relapse

A total of 12 functional definitions of relapse were identified, listed as follows: Hospitalization (all causes) (15 studies)^7^, ^17^, ^18^, ^19^, ^20^, ^21^, ^22^, ^23^, ^24^, ^25^, ^26^, ^27^, ^28^, ^29^, ^30^; Psychiatric‐related hospitalization (overall psychotic, mental/behavioral disorders) (7 studies)^20^, ^31^, ^32^, ^33^, ^34^, ^35^, ^36^; schizophrenia‐related hospitalizations (specific to symptoms of schizophrenia) (7 studies)^37^, ^38^, ^39^, ^40^, ^41^, ^42^, ^43^; schizophrenia‐related emergency department (ED) visits (3 studies)^39^, ^40^, ^41^; Psychiatric‐related ED visits (3 studies)^18^, ^29^, ^30^; Increase in the Clinical Global Impression Scale (CGI‐S) score (2 studies)^17^, ^44^ or according to psychiatrist assessment (1 study)^45^; Symptom re‐emergence (1 study)^20^; Intentional overdose events leading to ED visits or hospital admissions (1 study)^30^; Suicidality (including ideation and attempts) leading to ED visits or hospital admissions (1 study)^30^; ED visits (unspecified reasons) (1 study)^7^; Medication switch (1 study).^19^ Further details on methods of measurement of relapse may be found in Table 1.

**TABLE 1:** Criteria used to define and measure relapse in schizophrenia across retained publications

Definition	Measurement(s)
Hospitalization	Administrative claims^18^, ^21^, ^24^, ^27^, ^28^, ^29^
	EMRs^7^
	Medical chart review^17^, ^19^, ^24^, ^25^, ^26^, ^30^
	Medical chart review of inpatients in a behavioral health unit.^22^
	Medical chart review of indications for recurrence of significant psychotic symptoms, dangerous or violent behavior, or deteriorated functioning, without adequate response to outpatient treatment^23^
	Patient and clinician reports from mental health centers and VA hospitals^20^
Schizophrenia‐related hospitalization	Administrative claims^38^
	Administrative claims with mention of schizophrenia diagnosis^39^
	EMRs^40^, ^41^
	ICD‐9‐CM code 295.xx^37^
	National Discharge Register^42^, ^43^
Psychiatric‐related hospitalization	EMRs^35^
	ICD‐10 codes F00–F99^32^
	ICD‐10 codes F20–F29^31^
	Inpatient medical records from public psychiatric hospitals^34^
	Medical chart review^33^
	Medical chart review from general wards at psychiatric hospitals^36^
	Patient and clinician reports from mental health centers and VA hospitals^20^
Psychiatric‐related ED visits	Administrative claims^18^, ^29^
	Medical chart review^30^
Schizophrenia‐related ED visits	Administrative claims with mention of schizophrenia diagnosis^39^
	EMRs^40^
	ICD‐9‐CM code 295.xx^41^
Increase in CGI‐S Score	CGI increase of ≥1 point resulting in a score of ≥4^44^
	CGI increase of ≥2 points from the lowest CGI‐S score recorded^17^
Relapse based on psychiatrist assessment	Questionnaire^45^
Symptom re‐emergence	Patient and clinician reports from mental health centers and VA hospitals^20^
Intentional overdose leading to ED visit or hospital admission	Medical chart review^30^
Suicidality (ideation and attempts) leading to ED visit or hospital admission	Medical chart review^30^
ED visits	EMRs^7^
Medication switch	Medical chart review^19^

Abbreviations: CGI‐S, Clinical Global Impression Scale; ED, emergency department; EMRs, electronic medical records; ICD‐10, International Classification of Diseases, 10th Revision; ICD‐9‐CM, International Classification of Diseases, 9th Revision, Clinical Modification; VA, Veterans Health Administration.

Depending on the study, the same definition was based on different data sources and measurements. For example, hospitalization was either measured using administrative claims,^18^, ^21^, ^24^, ^27^, ^28^, ^29^ electronic medical records (EMRs),^7^ medical charts,^17^, ^19^, ^22^, ^23^, ^24^, ^25^, ^26^, ^30^ or reports from mental health centers and hospitals from integrated healthcare systems such as the Veterans Affairs (VA) in the United States (US).^20^ Schizophrenia‐related hospitalization was measured using administrative claims^38^, ^39^ based on diagnostic codes ICD‐9‐CM 295.0–295.9,^37^ national discharge registers,^42^, ^43^ and EMRs.^40^, ^41^ Likewise, psychiatric‐related hospitalizations were identified through EMRs,^35^ national patient registers using ICD‐10 codes F00–F99 and F20–F29,^31^, ^32^ or medical chart reviews from psychiatric hospitals and mental health centers.^20^, ^33^, ^34^, ^36^, ^44^ Another example is seen with the increase in CGI‐S scores for which thresholds used to define relapse differed from one study to the other.^17^, ^44^ These findings highlight the need to consider both definition of relapse as well as method of measurement.

Many definitions were used in combination with hospitalization to define relapse. For example, ED visits were combined with either hospitalization (*n* = 4; 11.4%)^7^, ^18^, ^29^, ^30^ or schizophrenia‐related hospitalization (*n* = 3; 8.6%).^39^, ^40^, ^41^ Similarly, symptom re‐emergence, suicidality, overdose events, and medication switch were all used as components to hospitalization to define relapse in schizophrenia (*n* = 3; 8.6%).^19^, ^20^, ^30^ Of note, no operational definition of relapse was provided in five publications.^46^, ^47^, ^48^, ^49^, ^50^

### 3.3 Handling of death

Death handling was only documented in 6 (17.1%) of the 35 studies,^17^, ^21^, ^31^, ^32^, ^42^, ^43^ and was considered as a study outcome in three studies.^32^, ^42^, ^43^ The first was a nationwide prospective cohort study conducted in Sweden between 2006 and 2013. Death, distinguished from suicide attempts, was assessed as part of a composite outcome of treatment failure.^32^ In two studies from Finland (one retrospective cohort and one prospective cohort), the risk of all‐cause death was a main outcome of interest.^42^, ^43^ Of note, in all three studies, death was not an outcome associated with schizophrenia relapse. In other cases, death was considered as a censoring criterion (2 studies)^17^, ^31^ or an exclusion criterion in one retrospective cohort study.^21^

### 3.4 Handling of loss to follow‐up

The handling of loss to follow‐up was specified in a minority of publications (10 studies): half used loss to follow‐up as censoring criterion,^17^, ^20^, ^25^, ^33^, ^43^ while the other half excluded patients lost to follow‐up.^7^, ^18^, ^24^, ^29^, ^44^ The quantitative impact of handling of loss to follow‐up on the comparative effectiveness estimates could not be assessed in this review, owing to the small number of studies. However, it is a known contributor to heterogeneity across observational studies.^51^

### 3.5 Heterogeneity of comparative effectiveness estimates

#### 3.5.1 Comparative effectiveness estimates

Among the 35 publications retained in this review, 13 (37.1%) reported comparative effectiveness estimates of LAIs versus OAPs. The results and characteristics for each study are summarized in Table 2. Based on an assessment of methodological heterogeneity, studies reporting HRs, RRs and ORs were considered separately. For sources reporting HR estimates, studies with differing follow‐up periods (1‐year and 2‐year) were analyzed separately.

**TABLE 2:** Overview of studies included in the assessment of heterogeneity of comparative effectiveness estimates of LAIs versus OAPs in relapse prevention (*n* = 13)

Reference	Country(ies)	Study design	Study period	Data source	Sample size	Age distribution (years)	Follow‐up		Relapse definition	Adjusted estimate (95% CI)
Brnabic et al. (2011)^17^	Australia, Mexico, Romania, Taiwan	Prospective cohort	Apr 2007–Jul 2009	Chart review	80	Median (LAI): 33 Median (OAP): 38	2 years	Hospitalization or increase in CGI‐S scale score by ≥2 points from the lowest CGI‐S score recorded		LAI HR: 0.17 (0.02–1.38)
Chang et al. (2012)^18^	Taiwan	Mirror‐image	Jan 2007–Dec 2007	Administrative claims	184	Mean: 41.5	12 months	Hospitalization and/or ED visits		SGA LAI RR: 0.56 (0.44–0.71)
Lafeuille et al. (2013)^41^	United States	Retrospective cohort	2006–2010	EMRs	3828	Mean (LAI): 42.1 Mean (OAP): 42.4	Mean: 30 months	Schizophrenia‐related hospitalization and/or ED visits		*Hospitalization:* SGA LAI HR: 0.88 (0.82–0.95) *ED visits:* SGA LAI HR: 0.99 (0.92–1.05)
Lafeuille et al. (2015)^40^	United States	Retrospective cohort	Jan 2009‐Mar 2012	EMRs	45 625	Mean (LAI): 41.4 Mean (OAP): 45.6	12 months	Schizophrenia‐related hospitalization and/or ED visits		SGA LAI HR: 0.68 (0.66–0.71)
Lin et al. (2019)^23^	Taiwan	Retrospective cohort	2006–2017	Chart review	12 169	Mean: 43.1	12 months	Hospitalization indicated for recurrence of significant psychotic symptoms, dangerous or violent behavior, or deteriorated functioning, without adequate response to outpatient treatment		LAI HR: 0.83 (0.78–0.88)
Marcus et al. (2015)^37^	United States	Retrospective cohort	Jan 2010–Jul 2013	Administrative claims	3768	Mean (LAI): 37.5 Mean (OAP): 38.0	6 months	Schizophrenia‐related hospitalization		LAI OR: 0.73 (0.54–0.99) FGA LAI OR: 1.01 (0.60–1.68) SGA LAI OR: 0.57 (0.37–0.88)
Novick et al. (2012)^33^	Denmark, France, Germany, Ireland, Italy, The Netherlands, Portugal, Spain, United Kingdom	Prospective cohort	2012*	Chart review	431	Mean (LAI): 40.4 Mean (OAP): 40.3	Max: 18 months	Psychiatric hospitalization		FGA LAI OR: 0.50 (0.27–0.93)
Shah et al. (2018)^28^	United States	Retrospective cohort	2010–2015	Administrative claims	22 490	Mean (LAI): 37.3 Mean (OAP): 37.0	12 months	Hospitalization		LAI HR: 0.66 (0.51–0.80)
Taipale et al. (2018)^31^	Finland	Retrospective cohort	1972–2015	Administrative claims	8719	Mean: 36.2	Median: 10.1 years	Psychiatric hospitalization		FGA LAI HR: 0.46 (0.4–0.54) SGA LAI HR: 0.45 (0.39–0.52)
Tiihonen et al. (2006)^43^	Finland	Prospective cohort	1995–2001	Administrative claims	2230	Mean: 30.7	Mean: 3.6 years	Schizophrenia‐related hospitalization		FGA LAI RR: 0.32 (0.22–0.49)
Tiihonen et al. (2011)^42^	Finland	Retrospective cohort	2000–2007	Administrative claims	2588	Mean: 37.8	Mean: 2 years	Schizophrenia‐related hospitalization		LAI HR: 0.36 (0.17–0.75) SGA LAI HR: 0.94 (0.55–1.63)
Tiihonen et al. (2017)^32^	Sweden	Prospective cohort	Jul 2006‐Dec 2013	Administrative claims	29 823	Mean: 44.9	Mean: 5.7 years	Psychiatric hospitalization		LAI HR: 0.78 (0.72–0.84) FGA LAI HR: 0.83 (0.75–0.92)
Voss et al. (2015)^39^	United States	Retrospective cohort	2006–2011	Administrative claims	218	Mean (LAI): 40.04 Mean (OAP): 42.2	12 months	Schizophrenia‐related hospitalization and/or ED visits		SGA LAI HR: 0.54 (0.32–0.92)

Abbreviations: CGI‐S, Clinical Global Impressions Scale; CI, confidence interval; ED, emergency department; EMR, electronic medical record; FGA, first‐generation antipsychotic; HR, hazard ratio; LAI, long‐acting injectable; OAP, oral antipsychotic; OR, odds ratio; RR, relative risk; SGA, second‐generation antipsychotic.

#### 3.5.2 Overall heterogeneity of studies with a 1‐year follow‐up (4 studies^23^, ^28^, ^39^, ^40^)

The *I* ^2^ statistic of HR estimates was 91%, exceeding the acceptable threshold for pooling (forest plot depicted in Figure S1). In the presence of such considerable heterogeneity, subgroups were further investigated based on relapse definition.

#### 3.5.3 Schizophrenia‐related hospitalization and/or ED visits (*n* = 2 studies^39^, ^40^)

Owing to the lack of heterogeneity (*I* ^2^ = 0%; *p* = .39), estimates were pooled resulting in a HR of 0.68 (95% CI, 0.66–0.70) (Figure S2). Both studies focused on second‐generation LAIs, specifically, paliperidone palmitate. However, it is important to note the weight attributed to each study, which might also explain the absence of heterogeneity. Evidently, the estimate obtained from the larger study (*n* = 45 625)^39^ dominates the findings from the other study (*n* = 218). ^40^

#### 3.5.4 Hospitalization as an indicator of schizophrenia relapse (*n* = 2 studies^23^, ^28^)

Substantial heterogeneity was observed (*I* ^2^ = 65%; *p* = .09), hence pooling was not recommended (Figure S3). Of note, hospitalization was measured differently from one study to another. In the retrospective cohort study conducted by Lin et al., 2019, medical charts were reviewed to identify hospitalizations indicated for the recurrence of significant psychotic symptoms, dangerous/violent behavior, or deteriorated functioning, without adequate response to outpatient treatment.^23^ On the other hand, the retrospective cohort study conducted by Shah et al., 2018 utilized administrative claims.^28^ In addition, comparisons were made using overall LAIs, rather than focusing on a specific type.

#### 3.5.5 Overall heterogeneity of studies with a 2‐year follow‐up (*n* = 3 studies^17^, ^41^, ^42^)

The heterogeneity of estimates originating from three studies that followed patients over a 2‐year period was acceptable (*I* ^2^ = 50%; *p* = .14). Consequently, the pooled HR estimate was 0.71 (95% CI, 0.43–1.16) (Figure S4). Although 50% is an acceptable threshold for heterogeneity, Cochrane guidelines indicate that an I^2^ between 30% and 60% may nonetheless represent moderate heterogeneity.^52^ Therefore, a subgroup analysis was conducted in an attempt to further reduce heterogeneity. In two studies that defined relapse as *schizophrenia*‐*related hospitalization*,^41^, ^42^ heterogeneity decreased to 42% (Figure S5). The corresponding pooled estimate was not statistically significant (HR = 0.80; 95% CI, 0.56–1.14). In this analysis, more weight was attributed to the larger study (*n* = 3828)^41^ than the other study (*n* = 2588).^42^

#### 3.5.6 Studies reporting RR estimates (*n* = 2 studies^18^, ^43^)

Substantial heterogeneity (*I* ^2^ = 83%; *p* = .01) was observed (Figure S6). Both studies, equally weighted, used different definitions of schizophrenia relapse. In a mirror‐image study, administrative claims were utilized to identify cases of relapse defined as hospitalization and/or ED visit.^18^ In a prospective cohort study from Finland, data from a national discharge register were utilized to identify schizophrenia‐related hospitalization as an indicator of relapse.^43^

#### 3.5.7 Studies reporting ORs (*n* = 2 studies^33^, ^37^)

The heterogeneity was substantial (*I* ^2^ = 66%; *p* = .09) (Figure S7). Each study defined and measured relapse differently. The first study defined relapse as schizophrenia‐related hospitalization according to ICD‐9‐CM codes 295.xx.^37^ The other study used medical charts to identify psychiatric‐related hospitalizations.^33^ A subgroup analysis according to relapse definition was not possible in this case, owing to the small number of studies included (*n* = 2).

## 4 DISCUSSION

This systematic review confirms that there is no standard method to define or measure schizophrenia relapse in observational studies, which is a major contributor to heterogeneity in estimates of comparative effectiveness of LAIs versus OAPs. Based on this review, 12 definitions were identified, and in studies with similar definitions, methods of measurement often differed. Similar to findings from the prior systematic review of observational studies,^1^ the current review found that hospitalization was the most common measure. In fact, this criterion was used in most publications either alone or as a component with other defining criteria. According to Olivares et al., hospitalization is often used to define relapse as it is a simple measure that provides tangible data.^1^ However, it is important to consider that the threshold for hospitalization may vary and reflect the healthcare system in specific countries or during specific time periods.^53^ In addition, not all clinically significant exacerbations of symptoms or relapses will result in hospitalization, and therefore this defining criterion might provide a limited view of potential differences in outcomes associated with the use of LAIs compared to OAPs.^37^ Unlike Olivares et al., this review did not identify a frequent use of clinical scales to define relapse in the real‐world setting.^1^ However, when studies used clinical scales, such as the CGI‐S scale, a variation in the cut‐off scores was observed.

According to the quantitative analysis, in studies that defined relapse as schizophrenia‐related hospitalization and/or ED visits over a 1‐year follow‐up period, LAIs were reported to be significantly more effective than OAPs in preventing relapse. LAIs also appeared to be advantageous in studies with 2‐year follow‐up periods that defined relapse as schizophrenia‐related hospitalization, however, not statistically significant. For studies based on hospitalization as a measure of schizophrenia relapse, heterogeneity was too high to allow pooling; yet this definition is the most frequently found in pooled estimates published in the literature.

Following this review, there remains a paucity of data on how death is handled in observational studies that compare the effectiveness between LAIs and OAPs among patients with schizophrenia. When reported, death was mostly assessed as a study outcome, independent of relapse‐related outcomes. According to the literature, schizophrenic patients face a mortality risk more than double that of the general population due to the high rates of suicide as well as comorbid cardiovascular disease, metabolic disorders, and infectious diseases.^36^ The lack of data reporting on death may underestimate relapse rates as episodes of relapse can be associated with suicide.^10^ Only one study included suicidality as a component of their definition for relapse, focusing on suicidal ideation and attempts, rather than deaths.^30^

Handling of loss to follow‐up was under‐reported in the observational studies retained. Of important note, variations in methods used for handling missing data due to loss to follow‐up pose a great challenge in meta‐analyses of observational studies as they lead to greater heterogeneity.^51^

This review has several limitations affecting the interpretation of results. First, a methodological quality assessment of retained studies was not performed, which would have allowed to establish the internal validity and risk of bias of included studies.^54^ Second, schizophrenia severity was not reported in most studies, as was the case in a meta‐analysis of mirror‐image studies conducted by Kishimoto et al., 2013.^53^ Severity of symptoms and cognitive defects might influence the effectiveness of LAIs, and therefore the inclusion of patients with different severities might also lead to heterogeneity across studies.^53^ Finally, the small number of studies that reported comparative effectiveness estimates between LAIs and OAPs restricts conclusions that may be drawn from meta‐analyses. Specifically, a meta‐regression to evaluate the concurrent effect of multiple potential sources of heterogeneity could not be performed, as a minimum of 10 studies is required. In addition, while the *I* ^2^ statistic is independent of the number of studies, it is found to be imprecise in small meta‐analyses,^55^ further complicating the assessment of heterogeneity. The weight attributed to each study also impacts pooled estimates. Studies with disproportionally larger sample sizes tend to have more precise results due to a smaller standard error and dominate the findings from smaller studies which may contribute to a lack of heterogeneity observed and is often a challenge found in pooling observational studies.^52^ The limited number of studies retained for the meta‐analysis also rendered the assessment of publication bias unfeasible, as statistical power of Egger's test may be too low to distinguish between chance asymmetry and real asymmetry when the number of studies is less than 10.^56^ Thus, a conclusion on whether the definition and measure of relapse impacted the heterogeneity cannot be drawn in this instance.

This study, however, presents several strengths. It offers a comprehensive synthesis of the various definitions and methods of measurement of relapse that have been used in observational studies. To our knowledge, this study is a first attempt to formalize the importance of considering the measurement of relapse when attempting to pool estimates that originate from various studies. Through tangible and recognized quantitative measures (I^2^ statistic), this review questions pooling of comparative effectiveness estimates based on different relapse definitions, as it has been done in previous meta‐analyses.

Findings from this review suggest that the variation in relapse definitions is a main source of heterogeneity in comparative effectiveness studies of schizophrenia treatment, which hampers the synthesis of evidence on treatment effectiveness. This calls for efforts to attempt to standardize the case ascertainment of schizophrenia relapse in observational studies.

## DISCLOSURE

All authors are employees of YolaRx Consultants that provides consultancy services to pharmaceutical companies, some of which are drug manufacturers of schizophrenia treatment, but not related to the work presented in this paper. No other conflicts of interest to declare.

## AUTHOR CONTRIBUTIONS

TC developed the protocol and conducted all activities related to the systematic review (searches, data extraction, meta‐analysis) as well as manuscript preparation. GC was the second independent reviewer for some of the searches and data extraction, and contributed to the meta‐analysis. JFN was responsible for the assessment of clinical and statistical heterogeneity. YM supervised and provided oversight for all components of the project, including the manuscript. All authors reviewed and approved the final version of the manuscript.

## ETHICS APPROVAL STATEMENT

As this was a systematic review, no ethics approval was sought.

## Supporting information

Figure S1–S7

## Data Availability

Data sharing is not applicable to this article as no new data were created or analyzed in this study.
