# Supplementary material for: Impact of schizophrenia relapse definition on the comparative effectiveness of oral versus injectable antipsychotics: A systematic review and meta‐analysis of observational studies
Source: Pharmacol Res Perspect. 2022 Jan 28;10(1):e00915. doi: 10.1002/prp2.915 (PMC8929363; doi:10.1002/prp2.915)
Supplement: Supplementary file 1 — Figure S1–S7 [file PRP2-10-e00915-s001.docx]

Supplementary Figure 1. Meta-analysis of effectiveness of LAIs versus OAPs in time to relapse, with 1-year follow-up (random effects model)

Supplementary Figure 2. Meta-analysis of effectiveness of LAIs versus OAPs in time to relapse, with 1-year follow-up, and relapse defined as schizophrenia-related hospitalization/ED visits (random effects model)

Supplementary Figure 3. Meta-analysis of effectiveness of LAIs versus OAPs in time to relapse, with 1-year follow-up, and relapse defined as hospitalization (random effects model)

Supplementary Figure 4. Meta-analysis of effectiveness of LAIs versus OAPs in time to relapse, with 2-year follow-up (random effects model)

Supplementary Figure 5. Meta-analysis of effectiveness of LAIs versus OAPs in time to relapse, with 2-year follow-up, and relapse defined as schizophrenia-related hospitalization (random effects model)

Supplementary Figure 6. Meta-analysis of effectiveness of LAIs versus OAPs measured by RR (random effects model)

Supplementary Figure 7. Meta-analysis of effectiveness of LAIs versus OAPs measured by OR (random effects model)
